# Pattern of population structuring between Belgian and Estonian bumblebees

**DOI:** 10.1038/s41598-019-46188-7

**Published:** 2019-07-04

**Authors:** Kevin Maebe, Reet Karise, Ivan Meeus, Marika Mänd, Guy Smagghe

**Affiliations:** 1 Department Plants and Crops, Faculty of Bioscience Engineering, Ghent University, Coupure links 653, B-9000 Ghent, Belgium; 2 Institute of Agricultural and Environmental Sciences, University of Life Sciences, Tartu, Estonia

**Keywords:** Genetic variation, Population dynamics, Conservation biology

## Abstract

Several population genetic studies investigated the extent of gene flow and population connectivity in bumblebees. In general, no restriction in gene flow is considered for mainland populations of common bumblebee species. Whether this assumption holds true for all species is not known. An assessment of bumblebee genetic structure in the context of their geographic distribution is needed to prioritize conservation and management needs. Here, we conducted a genetic study on seven bumblebee species occurring in Belgium and Estonia. Using 16 microsatellite markers, we investigated genetic diversity and population structuring in each species. This is the first study investigating population structuring of both declining and stable bumblebee species on both small and large geographic scales. Our results showed no or only low population structuring between the populations of the restricted and declining bumblebee species on both scales, while significant structuring was found for populations of the common species on the larger scale. The latter result, which may be due to human or environmental changes in the landscape, implies the need for the conservation of also widespread bumblebee species. Conservation strategies to improve gene flow and connectivity of populations could avoid the isolation and future losses of populations of these important species.

## Introduction

Bumblebees are essential pollinators for natural and managed ecosystems^1,2^. They experience worldwide declines, even more severe than many other pollinator species^3–5^. Different hypotheses aim to explain the observed declines in bee populations^3,4,6–8^. Although decline of bumblebee populations is clearly a multi-factorial phenomenon^7^, agricultural intensification, with increasing loss of habitats and plant species providing key forage resources, has been declared to be the key driver of the observed bumblebee declines across Europe^7–9^. Genetic factors play herein also a role. As genetic variability reflects a species potential to adapt to current and future changes in the environment, having a low genetic diversity will increase the likelihood towards extinction^9–12^. Genetic drift, founder effects, and inbreeding can all decrease genetic variability within populations^9,10,13^.

Gene flow can also alter the present levels of genetic diversity^9,10,14^. On one hand limited dispersal could lead to significant substructure and isolation of previously well-connected populations following a pattern in which nearby populations will be genetically more similar than those far away^15,16^. On the other hand high dispersal rates can buffer drift effects and lead to weak population substructure or even panmixia over large areas^9,10,14,17,18^.

In eusocial species, population structuring can occur due to differential dispersal rates between both sexes^19^. In bumblebees, queens and drones (males) are the only reproductive stages^20,21^. Although some indirect information on male and queen dispersal capabilities is available (e.g.^22,23^), accurate species specific data are currently lacking. However, dispersal and gene flow are key determinants of a species’ ability to respond to land-use change. Populations of bumblebee species with a more limited dispersal rate will have less chance of successfully recolonizing a suitable habitat and will be more vulnerable to inbreeding^9,10,13^. Furthermore, the amount of reproductive offspring a species produces will also contribute to the level of gene flow, as more reproductives may enlarge the chance of successful matings and exchange of genetic material between populations (as also discussed in^24^).

Geographical barriers, such as water bodies and mountains, can also greatly limit dispersal and gene flow^25^. Such barriers can block dispersal and thus limits gene flow between populations, leading to a non-uniform increase in genetic differentiation across the landscape^16^. In North America, population genetic studies have compared common and declining species within shared landscapes. These studies showed the impact of such geographic barriers, as they reported an increased population genetic differentiation for mountain and island species^12,17,23–30^, and a significant fine-scale spatial genetic structure for *B*. *vosnesenskii*^30^ due to human altered-landscapes^17^. For Europe, only studies investigating island populations of declining and common species in the UK described genetic differentiation and low levels of gene flow^31–35^. Studies with common species (*B*. *terrestris* and *B*. *pascuorum*) showed no population structuring on a European scale^36,37^ (respectively). Furthermore, Dreier *et al*.^38^ found only very low fine-scale spatial genetic structuring in two common species (*B*. *terrestr*is and *B*. *pascuorum*) out of the five investigated bumblebee species (two more common species: *B*. *hortorum*, *B*. *lapidarius* and one declining species: *B*. *ruderatus*). Thus, in general no restriction in gene flow is considered for mainland populations for most bumblebee species^39^. Whether this assumption holds true for all bumblebee species is not known. Although few population genetic studies have compared common and declining species within shared landscapes in Europe^18,38^, none have studied their population structure on both small and larger geographic scales. However, an assessment of bumblebee genetic structure in the context of their geographic distribution is needed to help prioritize conservation and management needs (as also stated by^12^).

**Figure 1:**
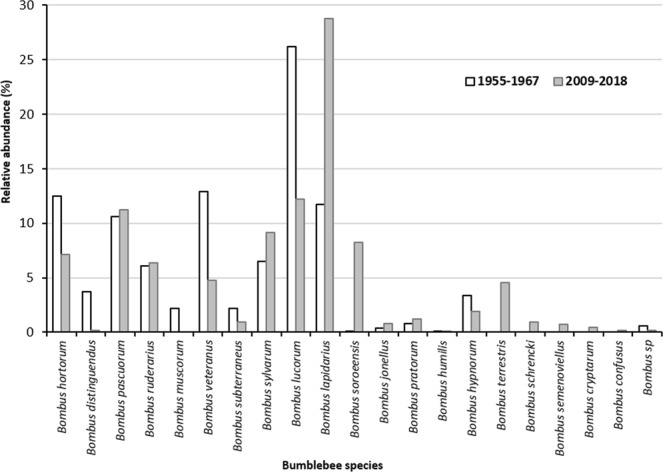
Bumblebee relative abundance in North-Central Estonia from 1955–1967 *versus* 2009–2018.

Here, we conducted a genetic study of seven social species of bumblebees occurring in Belgium and Estonia (*B*. *ruderarius*, *B*. *soroeensis*, *B*. *sylvarum*, *B*. *hortorum*, *B*. *hypnorum*, *B*. *lapidarius*, and *B*. *pascuorum*). Three species are considered to be declining and restricted in Belgium (*B*. *ruderarius*, *B*. *soroeensis*, and *B*. *sylvarum*), while the other four species have a nationally common and widespread distribution (*B*. *hortorum*, *B*. *hypnorum*, *B*. *lapidarius*, and *B*. *pascuorum*)^18^. Species status and distribution are different in Estonia. Indeed, when comparing 1955–1967 with 2009–2018 abundance data from the same area (North-central Estonia) increasing population trends were visible for *B*. *sylvarum*, *B*. *soroeensis* and *B*. *lapidarius*, while *B*. *hortorum* and *B*. *ruderarius* show clear negative trends (Fig. 1)^40,41^. Using 16 microsatellite markers, we investigated genetic diversity and population structuring by collecting bumblebee workers from each species at several locations, five in Belgium (Moorsel, Trivières, Francorchamps, Nieuwpoort and Torgny) and two in Estonia (Harjumaa and Põlvamaa) during bumblebee foraging seasons of 2013–2015 and 2015–2017, respectively. This approach allowed us to test our hypotheses that (i) no population structuring is present for populations of common bumblebee species, while (ii) limited gene flow is expected between populations of restricted and declining species. For the latter hypothesis, gene flow might not be limited at small spatial scale (between the two selected locations of Estonia or Belgium) but we expect it to be at least present at large scale due to less population connectivity, and fewer emerging queens. This is the first study investigating population structuring of both declining and stable bumblebee species on small and large spatial scale. This study has not only great impact on making species specific conservation strategies but also has major consequences for all studies performing population genomics with European bumblebee species as the assumption for the absence or present of structuring within species is a key factor for making an accurate sampling and experimental design.

## Results

All 16 microsatellites could be amplified and scored reliably in the seven *Bombus* species. After removal of full-sibs detected by Colony 2.0 and Kinalyzer analyses, and specimens with missing data at more than 5 out of 16 loci, 677 specimens remained for all further genetic analysis out of the 784 specimens (Table 1). No significant linkage disequilibrium between loci were detected. Testing for genotype frequencies against HW-expectations displayed no or only limited heterozygote deficits.

**Table 1:** Estimated mean *H*_E_ and *A*_R_ (±SE) for Belgian and Estonian populations of each bumblebee species.

Species	N	Location	Country	*H*_E_	SE	*A*_R_	SE
*B*. *hortorum*	25	Harjumaa	Estonia	0.546	0.088	3.01	0.36
*B*. *hortorum*	37	Polvamaa	Estonia	0.567	0.093	3.16	0.39
*B*. *hortorum*	25	Francorchamp	Belgium	0.587	0.092	3.23	0.38
*B*. *hortorum*	17	Trivières	Belgium	0.570	0.087	3.09	0.35
*B*. *hortorum*	19	Moorsel	Belgium	0.580	0.095	3.21	0.38
*B*. *hortorum*	23	Torgny	Belgium	0.550	0.093	3.06	0.38
*B*. *hypnorum*	3	Polvamaa	Estonia	0.329	0.090	1.94	0.27
*B*. *hypnorum*	8	Moorsel	Belgium	0.371	0.071	1.94	0.19
*B*. *hypnorum*	10	Torgny	Belgium	0.388	0.071	2.03	0.19
*B*. *lapidarius*	25	Harjumaa	Estonia	0.663	0.064	3.29	0.26
*B*. *lapidarius*	22	Polvamaa	Estonia	0.651	0.071	3.25	0.27
*B*. *lapidarius*	24	Nieuwpoort	Belgium	0.750	0.055	3.72	0.24
*B*. *lapidarius*	23	Francorchamp	Belgium	0.720	0.056	3.57	0.24
*B*. *lapidarius*	22	Trivières	Belgium	0.742	0.047	3.65	0.22
*B*. *lapidarius*	19	Moorsel	Belgium	0.719	0.054	3.57	0.24
*B*. *lapidarius*	21	Torgny	Belgium	0.739	0.054	3.66	0.25
*B*. *pascuorum*	24	Harjumaa	Estonia	0.445	0.087	2.53	0.32
*B*. *pascuorum*	33	Polvamaa	Estonia	0.443	0.084	2.50	0.32
*B*. *pascuorum*	19	Nieuwpoort	Belgium	0.410	0.086	2.47	0.35
*B*. *pascuorum*	26	Francorchamp	Belgium	0.456	0.085	2.56	0.32
*B*. *pascuorum*	23	Trivières	Belgium	0.436	0.083	2.46	0.31
*B*. *pascuorum*	21	Moorsel	Belgium	0.443	0.087	2.51	0.33
*B*. *pascuorum*	20	Torgny	Belgium	0.398	0.085	2.34	0.31
*B*. *ruderarius*	8	Harjumaa	Estonia	0.366	0.097	2.27	0.35
*B*. *ruderarius*	13	Polvamaa	Estonia	0.313	0.094	2.05	0.33
*B*. *ruderarius*	6	Nieuwpoort	Belgium	0.307	0.095	2.07	0.35
*B*. *ruderarius*	10	Torgny	Belgium	0.321	0.102	2.16	0.37
*B*. *soroeensis*	23	Harjumaa	Estonia	0.493	0.100	2.84	0.41
*B*. *soroeensis*	22	Polvamaa	Estonia	0.486	0.098	2.85	0.41
*B*. *soroeensis*	9	Torgny	Belgium	0.430	0.095	2.49	0.35
*B*. *sylvarum*	47	Harjumaa	Estonia	0.269	0.080	1.87	0.28
*B*. *sylvarum*	37	Polvamaa	Estonia	0.288	0.084	1.93	0.31
*B*. *sylvarum*	13	Torgny	Belgium	0.330	0.084	2.07	0.31

### Genetic diversity parameters *A*_R_ and *H*_E_

Overall populations and species, allelic richness (*A*_R_) ranged from 1.87 to 3.72, with a mean *A*_R_ of 2.71. Mean *H*_E_ was 0.488, with individual population values ranging from 0.269 to 0.750. For most species, *A*_R_ and *H*_E_ estimates were very similar overall populations, showing no significant differences between the Estonian and Belgian bumblebee populations (*B*. *hortorum*: *A*_R_, *t* = −1.206, *P* = 0.228; *H*_E_, *t* = −1.261, *P* = 0.207; *B*. *hypnorum*: *A*_R_, *t* = −0.334, *P* = 0.738; *H*_E_, *t* = −1.117, *P* = 0.264; *B*. *pascuorum*: *A*_R_, *t* = 0.937, *P* = 0.349; *H*_E_, *t* = 1.213, *P* = 0.225, and *B*. *ruderarius*: *A*_R_, *t* = 0.488, *P* = 0.626; *H*_E_, *t* = 1.201, *P* = 0.230; Table 1). However, in *B*. *lapidarius* and *B*. *sylvarum*, both genetic diversity parameters were significantly higher in Belgian populations (*B*. *lapidarius*: *A*_R_, *t* = −6.096, *P* < 0.001; *H*_E_, *t* = −5.290, *P* < 0.001, and *B*. *sylvarum*: *A*_R_, *t* = −2.145, *P* = 0.032; *H*_E_, *t* = −2.185, *P* = 0.029; Table 1), while for *B*. *soroeensis* only *A*_R_ was significantly higher in Estonian populations (*t* = −2.145, *P* = 0.032; Table 1).

### Population structuring within Bombus species

**Figure 2:**
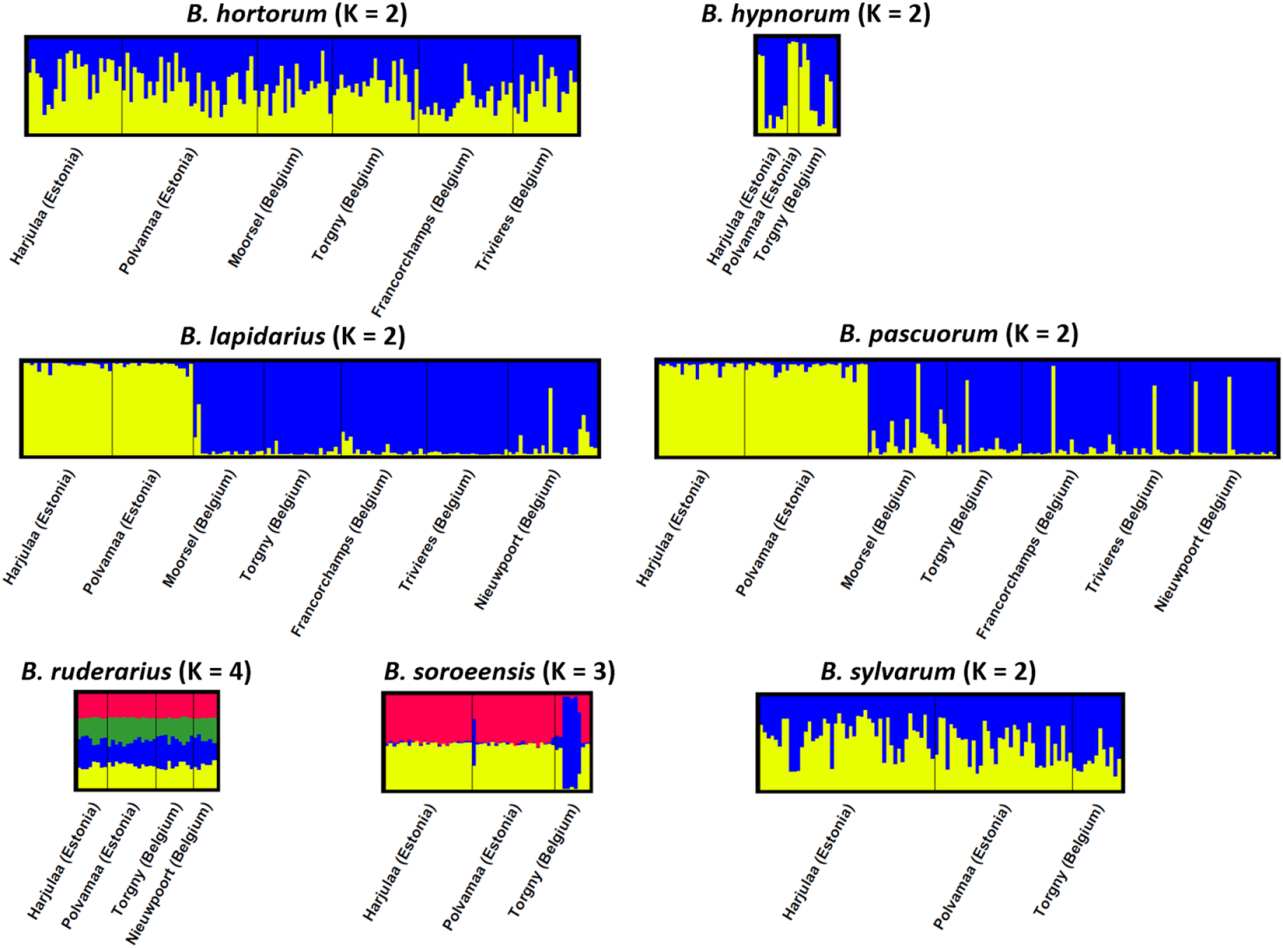
Bayesian clustering for each bumblebee species analysis. Each specimen is represented by a single bar, and assigned to a certain cluster by color. Specimens belonging to the same original population are grouped within black vertical lines.

Overall locations genetic differentiation was significant in *B*. *hypnorum* (*F*_ST_ = 0.107; Dest = 0.175; *P* < 0.001), *B*. *lapidarius* (*F*_ST_ = 0. 036; Dest = 0.128; *P* < 0.001) and in *B*. *pascuorum* (*F*_ST_ = 0.067; Dest = 0.120; *P* < 0.001). Global *F*_ST_ and Dest-estimates were low in the other four species (in *B*. *hortorum*, *F*_ST_ = 0.010, Dest = 0.024; in *B*. *ruderarius*, *F*_ST_ = 0.034, Dest = 0.050; in *B*. *soroeensis*, *F*_ST_ = 0.022, Dest = 0.043; in *B*. *sylvarum*, *F*_ST_ = 0.029, Dest = 0.041; all *P* < 0.001). Pairwise *F*_ST_ comparisons were significant (*P* < 0.05) for 46 of 72 comparisons ranging from 0.014–0.231 (Table S1). Most significant comparisons were between countries, while only two comparisons were low but significant present between the two Estonian locations (for *B*. *hortorum* and *B*. *sylvarum*, *F*_ST_ = 0.017 and *F*_ST_ = 0.016, respectively; Table S1), and eight pairwise *F*_ST_ were significant between Belgian locations (in *B*. *hortorum* between Torgny and Francorchamps, *F*_ST_ = 0.017; in *B*. *lapidarius* between Francorchamps and Nieuwpoort, *F*_ST_ = 0.017; and in *B*. *pascuorum* for all comparisons with Nieuwpoort, *F*_ST_-values from 0.023 to 0.046, between Torgny and Francorchamps, *F*_ST_ = 0.033, and between Torgny and Trivières, *F*_ST_ = 0.026; Table S2). Similar results were observed for pairwise Dest comparisons. The same 46 comparisons were significant (*P* < 0.05; Table S2) but in general Dest-values were higher within population pairs (Tables S1 and S2). Hence, based on two first principal components, the Principal Component Analysis plot showed a clear spatial pattern between Estonian and Belgian populations of *B*. *hypnorum*, *B*. *lapidarius*, and *B*. *pascuorum* (Fig. 2).

**Figure 3:**
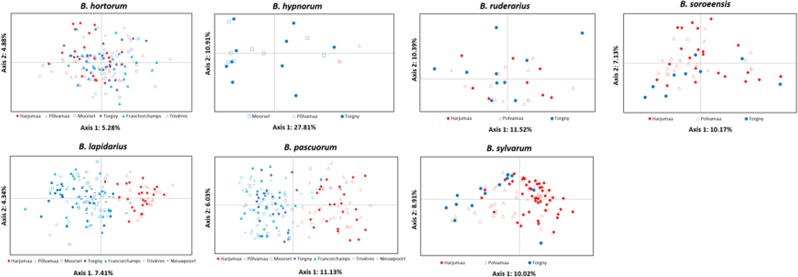
Principal Coordinates Analysis (PCoA) based on standardized genetic distance. Plot of the first two axes for *B*. *hortorum*, *B*. *hypnorum*, *B*. *lapidarius*, *B*. *pascuorum*, *B*. *ruderarius*, *B*. *soroeensis* and *B*. *sylvarum*. In red and blue, Estonian and Belgian locations.

By applying the Evanno method embedded in Structure Harvester, we identified K = 2 for almost all bumblebee species (*B*. *hypnorum*, *B*. *lapidarius*, *B*. *pascuorum*, *B*. *ruderarius* and *B*. *sylvarum*; Fig. A1). Furthermore, the best *K*-value for *B*. *hortorum* and *B*. *soroeensis* were identified as K = 4 and K = 3; respectively (Fig. A2). However, our Structure results showed no clear clustering at best identified number of populations for four species (*B*. *hortorum*, *B*. *ruderarius*, *B*. *soroeensis*, and *B*. *sylvarum*). Indeed, all specimens evenly belonged to each identified populations (Fig. 3). Furthermore, as the Evanno method is not able to calculate K = 1, we therefore changed K to 1, highlighting that no structuring is retrieved in these species. However for *B*. *hypnorum*, *B*. *lapidarius* and *B*. *pascuorum*, K remained at 2, showing population structuring between Belgian and Estonian populations (Fig. 3).

The populations of all *Bombus* species were also per species grouped in two clusters (K = 2) by Geneland analysis (Fig. A2). As both clusters contained only the Belgian locations or Estonian populations, these results showed population structuring between Belgian and Estonian populations.

## Discussion

Here, we investigated population structuring in three for Belgium declining and restricted bumblebee species (*B*. *ruderarius*, *B*. *soroeensis*, and *B*. *sylvarum*) and four more stable and widespread bumblebee species (*B*. *hortorum*, *B*. *hypnorum*, *B*. *lapidarius*, and *B*. *pascuorum*) on small and large spatial scale. Specifically, we tested the hypotheses that (i) population structuring is absent between populations of common bumblebee species on both scales, while (ii) between populations of restricted and declining species gene flow can be limited at the small scale and structuring should be present at the larger geographic scale. For the common bumblebee species we found no population structuring on a small scale but on the large scale population structuring was present in three out of the four common species (in *B*. *hypnorum*, *B*. *lapidarius* and *B*. *pascuorum*). That our data was not able to support our first hypothesis was rather unexpected considering previous studies showing no population structuring for mainland populations of North-American bumblebee species^29,39,42^, and for two common European species (*B*. *terrestris* and *B*. *pascuorum*) on an European scale^36,37^. Although in the latter species, some indications of low genetic differentiation between central European and Scandinavian populations were observed, significant differentiation was only present between populations below and above the Alps showing the impact of large geographic barriers on population structuring^37^. Indeed, significant genetic differentiation between mainland populations have been shown in several widespread species across natural barriers such as mountains^27,28,37^, and between island and mainland populations^17,26–28,35^. There were no great mountains present within our study design to limit gene flow, and although the Baltic Sea could be considered as a great water body possibly limiting gene flow, we believe that there are still enough possibilities for gene flow over the main European continent. Hence, as genetic structuring could be present at both low and continental scale due to human-modified landscapes^17,30,38^ this presumably caused the significant differentiation between Belgian and Estonian populations. Another possible explanation is demonstrated in *B*. *lapidarius* by Lecocq *et al*.^43^. These authors showed evidence of genetic allopatric differentiation in *B*. *lapidarius* caused by population movement during Quaternary climatic oscillations. Belgian and Estonian *B*. *lapidarius* may have found refuge in another of the main refugia (Iberian Peninsula, Balkans, Centre-Eastern Europe, and Southern Italy) during Ice Ages causing differentiation, and may be reinforced during post-glacial recolonization^43–45^. Although the hypothesis of different Ice-Age refugia has also been suggested in *B*. *pascuorum*^37^, more research is needed to support this hypothesis for both *B*. *hypnorum* and *B*. *pascuorum*, and to better understand the impact of Ice Age refugia on bumblebee population differentiation.

As our previous results demonstrated population differentiation in widespread bumblebee species on a large scale, we expected to observe greater or at least similar levels of population structuring in the restricted species. Indeed, due to the restricted distribution and declining population trends of these restricted species, one would expect limited gene flow due to less population connectivity, and fewer emerging queens. However, our result showed no or only very low structuring on both geographic scales between populations of restricted, declining bumblebee species. Maybe these restricted species have large dispersal abilities on mainland Europe, allowing them to provide sufficient gene flow between distant populations. However, as clear and accurate measurements of queen (and male) dispersal rates are currently lacking, this hypothesis cannot be tested.

It is important to realize that technical aspects may also influence our results of structuring within the restricted species. The low level of genetic diversity detected within the restricted species (*B*. *ruderarius* and *B*. *sylvarum;* see Table 1) could impair the ability to detect population structuring. Indeed, due to past population dynamics (as discussed in^18^) genetic diversity maybe became so low that no or only few rare alleles are present and thus additional losses due to drift and limited gene flow are unlikely to be detected. The absence of population structuring in restricted species may thus be an artifact of the limited amount of markers used within our study. Recent advances in genotyping-by-sequencing (GBS) methods such as RAD-sequencing (restriction site-associated DNA sequencing) made screening the bumblebee genome for thousands of polymorphisms possible^39,42–46^ allowing the possibility to greatly enlarge the power of population differentiation studies among others. Future research is needed to exclude possible biases due to marker choice.

Finally, our results can contribute to current conservation management of restricted bumblebees. The absence of genetic structure within the declining species suggest that the European agricultural landscape is no barrier for gene flow possibly due to queen dispersal. However, as the latter seems unlikely considering multiple studies showing low dispersal abilities within these species^26,31–35^, clear measurements of queen dispersal possibilities are needed. Furthermore, as these declining species are characterized with low levels of genetic diversity (see also Maebe *et al*.^18^) they are more vulnerable for population losses due to environmental changes, which may even lead to local extinction^9,10^. Therefore, populations of declining bumblebees should be monitored over the European mainland for their present genetic diversity levels. This knowledge will allow for the development of accurate conservation and management strategies to help maintain or increase current genetic diversity levels, current population connectivity and their effective population sizes. The presence of structuring in the populations of more widespread, stable species has also major impacts on their conservation. Although there is still a high genetic diversity present within their populations, changing landscapes due to human, environmental or climate changes may limit gene flow between populations of these common species. The connection between populations, which have belonged to one metapopulation, must be ensured to allow gene flow and avoid possible future genetic isolation. Furthermore, additional studies must enlarge our knowledge on how human-modified landscapes may influence population structuring^17,26,38^ and which role glacial refugia had on current bumble bee population differentiation.

## Material and Methods

### Sampling and proportional abundance measurement

**Figure 4:**
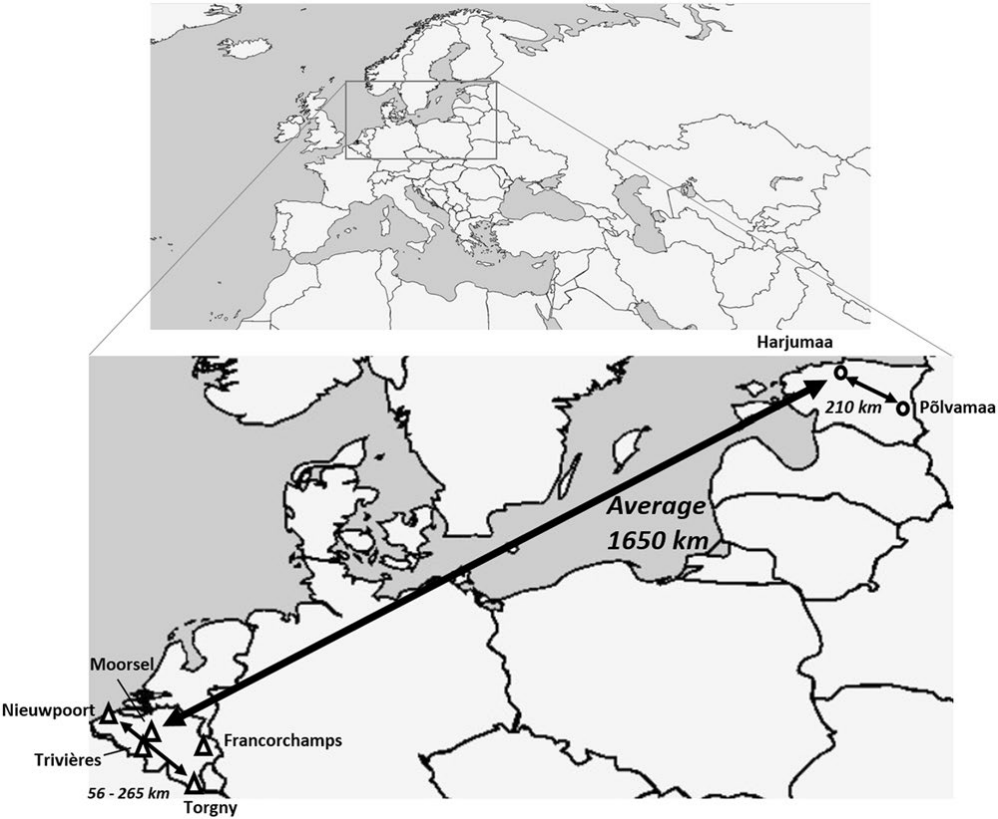
Map of sampling locations.

To be able to compare genetic diversity and population structuring at small and large geographic scale, we selected several sampling locations from two European countries (Belgium and Estonia, around 1.650 km separated; Fig. 4). In Belgium, Moorsel, Francorchamps, Trivières, Nieuwpoort and Torgny were selected as sampling locations, which are separated 56 to 265 km. A total of 439 workers of seven bumblebee species (*B*. *ruderarius*, *B*. *soroeensis*, *B*. *sylvarum*, *B*. *hortorum*, *B*. *hypnorum*, *B*. *lapidarius*, and *B*. *pascuorum*) were collected from these five locations. These specimens were already genotyped with 16 microsatellites to study temporal stability of genetic diversity^16^. All bumblebees were sampled during straight ahead transect walks using a net, and during suitable weather conditions for pollinators at three and four days during the bumblebee foraging season of 2013 and 2015, respectively. For the two Estonian locations; Harjumaa and Põlvamaa, which are separated 210 km, 345 specimens of the same seven bumble bee species were sampled during the bumblebee foraging seasons of 2015 and 2017 (Table 1). All specimens were collected individually with small glass jars from the flowers, killed within chloroform, and individually stored in the freezer at −20 °C awaiting further DNA extraction.

### DNA extraction and microsatellite protocol

DNA extraction of individual bumblebee workers were performed on one middle leg using a Chelex DNA extraction protocol as described in Maebe *et al*.^47^. Extractions were afterwards stored frozen at −20 °C. Each specimen was genotyped with 16 microsatellite (MS) loci. These 16 MS loci gave reliable signals in previous research using different bumblebee species^16,47^: BL13, BT02, BT23, BT24, BL02, BT04, BT05, BT08, and BT10^48^; B100, B11, B126, and B132^49^; and 0294, 0304, and 0810^50^; (Table S3). MS were amplified by multiplex PCR in 10 µl using the Type-it QIAGEN PCR kit. Each reaction contained 1.33 µl template DNA, Type-it Multiplex PCR Master Mix (2x, Qiagen) and the forward and reverse primer of four MS loci for each of four multiplex mixes as described in Maebe *et al*.^18^. The PCR protocol, and capillary electrophoreses on an ABI-3730xl sequencer (Applied Biosystems), were performed with the method as described in Maebe *et al*.^51^. The fragments were examined and scored manually using Peak Scanner Software v 2.0 (Applied Biosystems).

### Linkage disequilibrium, Hardy-Weinberg equilibrium, and sister detection

All populations were tested for genotypic linkage disequilibrium, deviations from Hardy-Weinberg equilibrium (HW) and the presences of null alleles using the program Fstat 2.9.3^52^, GenAlEx v6.5^53^, and Microchecker^54^, respectively. As described in Maebe *et al*.^47^, we removed all specimens which could not be scored in a reliable manner for at least 10 loci, and retained only one sister per colony by detecting full-siblings with the programs Colony 2.0^55^ employing corrections for genotyping errors (5% per locus), and by the 2 allele algorithm and consensus method implemented in Kinanalyzer^56^.

### Genetic diversity

Nei’s unbiased expected heterozygosity (*H*_E_) and the observed heterozygosity (*H*_O_)^57^ were determined with GenAlEx v6.5^53^ for all populations per species. Furthermore, we estimated the sample size-corrected private allelic richness (*A*_*R*_) with the program Hp-Rare 1.1^58^ normalized to 10 gene copies.

Differences in genetic diversity levels between countries were investigated per species by linear Mixed Models (LMMs). LMMs were performed for both *A*_R_ and *H*_E_ in RStudio^59^ with R package lme4 version 1.1–10^60^. The model included country as fixed factor and microsatellite loci as random factor to account for inter-locus variability^61,62^. R package multcomp was used to perform Tukey HSD post-hoc comparisons^62,63^.

### Population structuring

Population differentiation within each *Bombus* species was inferred by estimation of pairwise *F*_ST_ and *D*_est_^64^ with 999 permutations using Genalex v6.5^53^. Furthermore, Principal Coordinates Analysis (PCoA) were made using standardized genetic distance matrix to check for population substructuring.

Per species population structuring was also investigated by performing a Bayesian clustering algorithm imbedded in the software Structure v. 2.3.3^65^. The number of best fitting populations (K) was explored using the admixture model which was set with 500,000 burn-in steps and 1,000,000 samples. For each species, K-values ranged from 1 to 10, and were repeated 9 times. The best K-value was determined by the Evanno method^66^ imbedded in the program Structure Harvester v. 0.6.94^67^. A final bar plot for the best K-value was created by using the replicate structure runs at this K-value in CLUMPP^68^. Membership coefficients were depicted using Distruct v.1.1^69^.

Finally, population structure within each *Bombus* species was also inferred with another Bayesian clustering method, Geneland 4.0.6^70^. Geneland adds the effect of geographic location on the estimation of best fitted K-values, by estimation of K including the spatial coordinates into the model. Population assignment model, with correlated allele frequency and null allele correction, was run for K ranging from 1 to 10 with 1,000,000 iterations, 100 thinning and 1,000 burn-in, and a spatial coordinate uncertainty of one. All other parameters were set as default.

## Supplementary information

Supplementary datafiles

## Data Availability

Microsatellite genotypes of each specimen will be archived at DRYAD: 10.5061/dryad.p57v3b3.
