# Supplementary material for: Pattern of population structuring between Belgian and Estonian bumblebees
Source: Sci Rep. 2019 Jul 4;9:9651. doi: 10.1038/s41598-019-46188-7 (PMC6609714; doi:10.1038/s41598-019-46188-7)

Pattern of population structuring between Belgian and Estonian bumblebees

Kevin Maebe[1], Reet Karise[2], Ivan Meeus[1], Marika Mänd[2], and Guy Smagghe[1*]

[1]Department Plants and Crops, Faculty of Bioscience Engineering, Ghent University, Coupure links 653, B-9000, Ghent, Belgium

[2]Institute of Agricultural and Environmental Sciences, University of Life Sciences, Tartu, Estonia

* Corresponding author: kevin.maebe@ugent.be and guy.smagghe@ugent.be

Table S1. Pairwise population matrix of *F*st-values. *F*st-values are shown below the diagonal, and *P*-values based on 999 permutations above the diagonal. Significant *F*st-values are indicated in bold.

Species	Locations							
	Estonia		***Belgium***					
	Harjumaa	Põlvamaa	Moorsel	Torgny	Francorchamps	Trivières		
B. hortorum	-	0.001	0.008	0.009	0.001	0.001	Harjumaa	
	**0.017**	-	0.171	0.013	0.001	0.013	Põlvamaa	
	**0.020**	0.012	-	0.128	0.553	0.344	Moorsel	
	**0.018**	**0.014**	0.015	-	0.010	0.304	Torgny	
	**0.024**	**0.014**	0.012	**0.017**	-	0.655	Francorchamps	
	**0.025**	**0.017**	0.016	0.015	0.013	-	Trivières	
		Põlvamaa	Moorsel	Torgny				
B. hypnorum		-	0.007	0.011	Põlvamaa			
		**0.231**	-	0.465	Moorsel			
		**0.205**	0.039	-	Torgny			
	Harjumaa	Põlvamaa	Moorsel	Torgny	Francorchamps	Trivières	Nieuwpoort	
B. lapidarius	-	0.056	0.001	0.001	0.001	0.001	0.001	Harjumaa
	0.015	-	0.001	0.001	0.001	0.001	0.001	Põlvamaa
	**0.042**	**0.050**	-	0.237	0.176	0.249	0.090	Moorsel
	**0.050**	**0.058**	0.016	-	0.528	0.762	0.103	Torgny
	**0.047**	**0.053**	0.016	0.012	-	0.563	0.013	Francorchamps
	**0.047**	**0.054**	0.016	0.012	0.012	-	0.054	Trivières
	**0.037**	**0.043**	0.016	0.015	**0.017**	0.016	-	Nieuwpoort
	Harjumaa	Põlvamaa	Moorsel	Torgny	Francorchamps	Trivières	Nieuwpoort	
B. pascuorum	-	0.296	0.001	0.001	0.001	0.001	0.001	Harjumaa
	0.011	-	0.001	0.001	0.001	0.001	0.001	Põlvamaa
	**0.055**	**0.050**	-	0.200	0.070	0.121	0.001	Moorsel
	**0.085**	**0.074**	0.016	-	0.001	0.001	0.001	Torgny
	**0.068**	**0.065**	0.016	**0.033**	-	0.496	0.001	Francorchamps
	**0.070**	**0.062**	0.016	**0.026**	0.011	-	0.001	Trivières
	**0.088**	**0.082**	**0.033**	**0.046**	**0.023**	**0.030**	-	Nieuwpoort
	Harjumaa	Põlvamaa	Torgny	Nieuwpoort				
B. ruderarius	-	0.482	0.227	0.030	Harjumaa			
	0.031	-	0.002	0.007	Põlvamaa			
	0.045	**0.056**	-	0.111	Torgny			
	**0.072**	**0.076**	0.071	-	Nieuwpoort			
	Harjumaa	Polvamaa	Torgny					
B. soroeensis	-	0.053	0.001	Harjumaa				
	0.017	-	0.007	Polvamaa				
	**0.050**	**0.044**	-	Torgny				
	Harjumaa	Polvamaa	Torgny					
B. sylvarum	-	0.001	0.001	Harjumaa				
	**0.016**	-	0.044	Polvamaa				
	**0.045**	**0.024**	-	Torgny				

Table S2. Pairwise population matrix of Dest-values. Dest-values are shown below the diagonal, and *P*-values based on 999 permutations above the diagonal. Significant Dest-values are indicated in bold.

Species	Locations							
	Estonia			***Belgium***				
	Harjumaa	Põlvamaa	Moorsel	Torgny	Francorchamps	Trivières		
B. hortorum	-	0.001	0.008	0.009	0.001	0.001	Harjumaa	
	**0.020**	-	0.175	0.014	0.003	0.015	Põlvamaa	
	**0.019**	0.005	-	0.127	0.550	0.344	Moorsel	
	**0.016**	**0.012**	0.007	-	0.010	0.305	Torgny	
	**0.035**	**0.014**	0.001	**0.013**	-	0.659	Francorchamps	
	**0.028**	**0.015**	0.002	0.013	0.003	-	Trivières	
		Põlvamaa	Moorsel	Torgny				
B. hypnorum		-	0.007	0.005	Põlvamaa			
		**0.208**	-	0.468	Moorsel			
		**0.183**	0.001	-	Torgny			
	Harjumaa	Põlvamaa	Moorsel	Torgny	Francorchamps	Trivières	Nieuwpoort	
B. lapidarius	-	0.057	0.001	0.001	0.001	0.001	0.001	Harjumaa
	0.015	-	0.001	0.001	0.001	0.001	0.001	Põlvamaa
	**0.135**	**0.165**	-	0.236	0.176	0.243	0.093	Moorsel
	**0.188**	**0.217**	0.010	-	0.529	0.761	0.105	Torgny
	**0.165**	**0.185**	0.012	0.002	-	0.565	0.013	Francorchamps
	**0.172**	**0.201**	0.010	0.009	0.003	-	0.056	Trivières
	**0.128**	**0.151**	0.018	0.015	**0.031**	0.021	-	Nieuwpoort
	Harjumaa	Põlvamaa	Moorsel	Torgny	Francorchamps	Trivières	Nieuwpoort	
B. pascuorum	-	0.280	0.001	0.001	0.001	0.001	0.001	Harjumaa
	0.002	-	0.001	0.001	0.001	0.001	0.001	Põlvamaa
	**0.072**	**0.066**	-	0.201	0.070	0.119	0.001	Moorsel
	**0.115**	**0.099**	0.003	-	0.001	0.001	0.001	Torgny
	**0.100**	**0.096**	0.007	**0.031**	-	0.495	0.002	Francorchamps
	**0.098**	**0.086**	0.005	**0.019**	0.001	-	0.001	Trivières
	**0.125**	**0.116**	**0.029**	**0.046**	**0.016**	**0.025**	-	Nieuwpoort
	Harjumaa	Põlvamaa	Torgny	Nieuwpoort				
B. ruderarius	-	0.469	0.215	0.035	Harjumaa			
	0.001	-	0.003	0.007	Põlvamaa			
	0.008	**0.025**	-	0.108	Torgny			
	**0.023**	**0.031**	0.019	-	Nieuwpoort			
	Harjumaa	Polvamaa	Torgny					
B. soroeensis	-	0.053	0.002	Harjumaa				
	0.010	-	0.015	Polvamaa				
	**0.040**	**0.027**	-	Torgny				
	Harjumaa	Polvamaa	Torgny					
B. sylvarum	-	0.001	0.001	Harjumaa				
	**0.007**	-	0.048	Polvamaa				
	**0.027**	**0.007**	-	Torgny				

Table S3. List of each microsatellite primer and multiplex used. Forward and reverse primer sequences, fluorescent dyes, multiplex (MP), annealing temperature (*Ta*), original size range of the PCR product, location in *B. terrestris* genome (LG) and the original reference are given for each SSR marker.

Marker	MP	Dye	LG *B.t.*	Primer Sequence Forward	Primer Sequence Reverse	*Ta*	Original Range	Origin
BL13	1	PET	B15	CGAATGTTGGGATTTTCGTG	GCGAGTACGTGTACGTGTTCTATG	53	205-217	Reber-Funk et al., 2006
BT02	1	NED	B11	AGGAACCGAGCGATAGAACCAC	GCTTTGCCTTTCCATCTTGCTG	53	175-183	Reber-Funk et al., 2006
BT23	1	FAM	B11	GCAACAGAAAATCGTCGGTAGTG	GCGGCAATAAAGCAATCGG	54	198-216	Reber-Funk et al., 2006
BT24	1	VIC	B07	TCTTTCCGTTTTCCCCCTG	CACCCACTTACATACATACACGCTC	52	227-257	Reber-Funk et al., 2006
BL02	2	NED	B01	GAACAGTGAGAGCGAGGAACAGAG	TTGCCACGTATATCCGAGCGAACC	52	163-171	Reber-Funk et al., 2006
BT04	2	FAM	B13	GAGAGAGATCGAATGGTGAGAGC	TGAGCACGTTCTTTCGTTCAC	52	183-199	Reber-Funk et al., 2006
BT08	2	PET	B03	AGAACCTCCGTATCCCTTCG	AGCCTACCCAGTGCTGAAAC	52	208-230	Reber-Funk et al., 2006
BT10	2	VIC	B08	TCTTGCTATCCACCACCCGC	GGACAGAAGCATAGACGCACCG	53	178-188	Reber-Funk et al., 2006
B100	3	FAM	B03	CGTCCTCGTATCGGGCTAAC	CGTGGAAACGTCGTGACG	58	146-198	Estoup et al., 1995;1996
B11	3	NED		GCAACGAAACTCGAAATCG	GTTCATCCAAGTTTCATCCG	52	124-136	Estoup et al., 1995;1996
B126	3	PET	B08	GCTTGCTGGTGAATTGTGC	CGATTCTCTCGTGTACTCC	56	172-176	Estoup et al., 1995;1996
B132	3	VIC	B11	GAAATTCGTGCGGAGGG	CAGAGAACTACCTAGTGCTACGC	58	148-213	Estoup et al., 1995;1996
0294_10o4	4	FAM	B09	AGTACGATAAAGCCAGGAAAG	TGTATGCCTATTGTACGAGTGT	55	169-177	Stolle et al., 2011
0304_9i13	4	NED	B04	GTATGAGTGAGTGATGTGCAAG	CCCTTCATCTCTGAACAATATC	55	154-160	Stolle et al., 2011
0810_65a23	4	PET	B06	TTAACAAATCCGAATTTAAAGG	GATAGTGGTTGCTTGTCATCTT	55	136-140	Stolle et al., 2011
BT05	4	VIC	B02	TTTCCTATGCCGAACGTCACC	CCCAGATAAAAGACCGCCTCTAGTC	53	194-220	Reber-Funk et al., 2006

Fig. A1. ΔK calculated by the Evanno method for each *Bombus* species

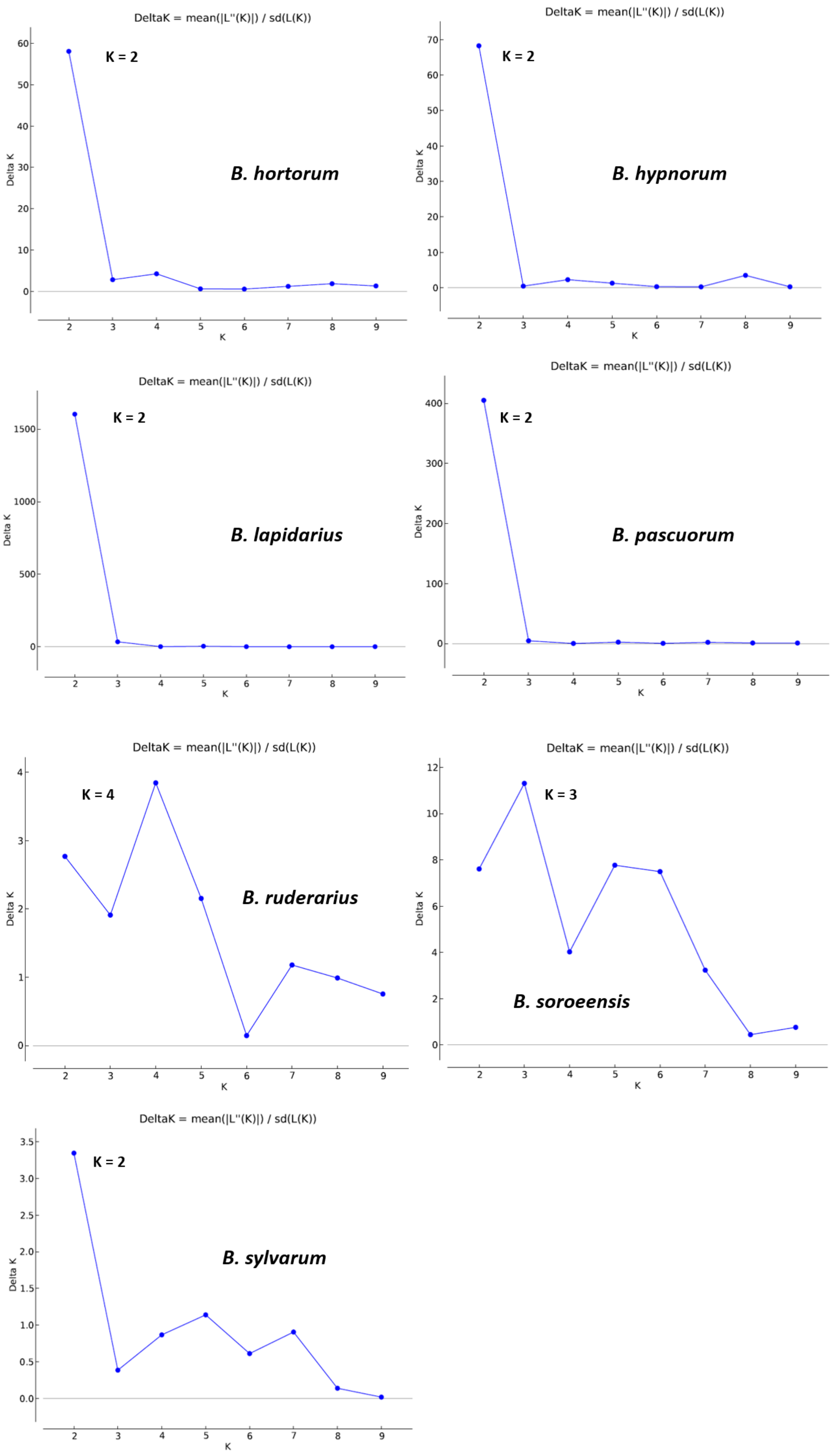

Fig. A2. Population clustering for each *Bombus* species estimated by Geneland analysis. Each map represents a detected cluster with population membership indicated in color: a high membership = light yellow; a low membership = dark red

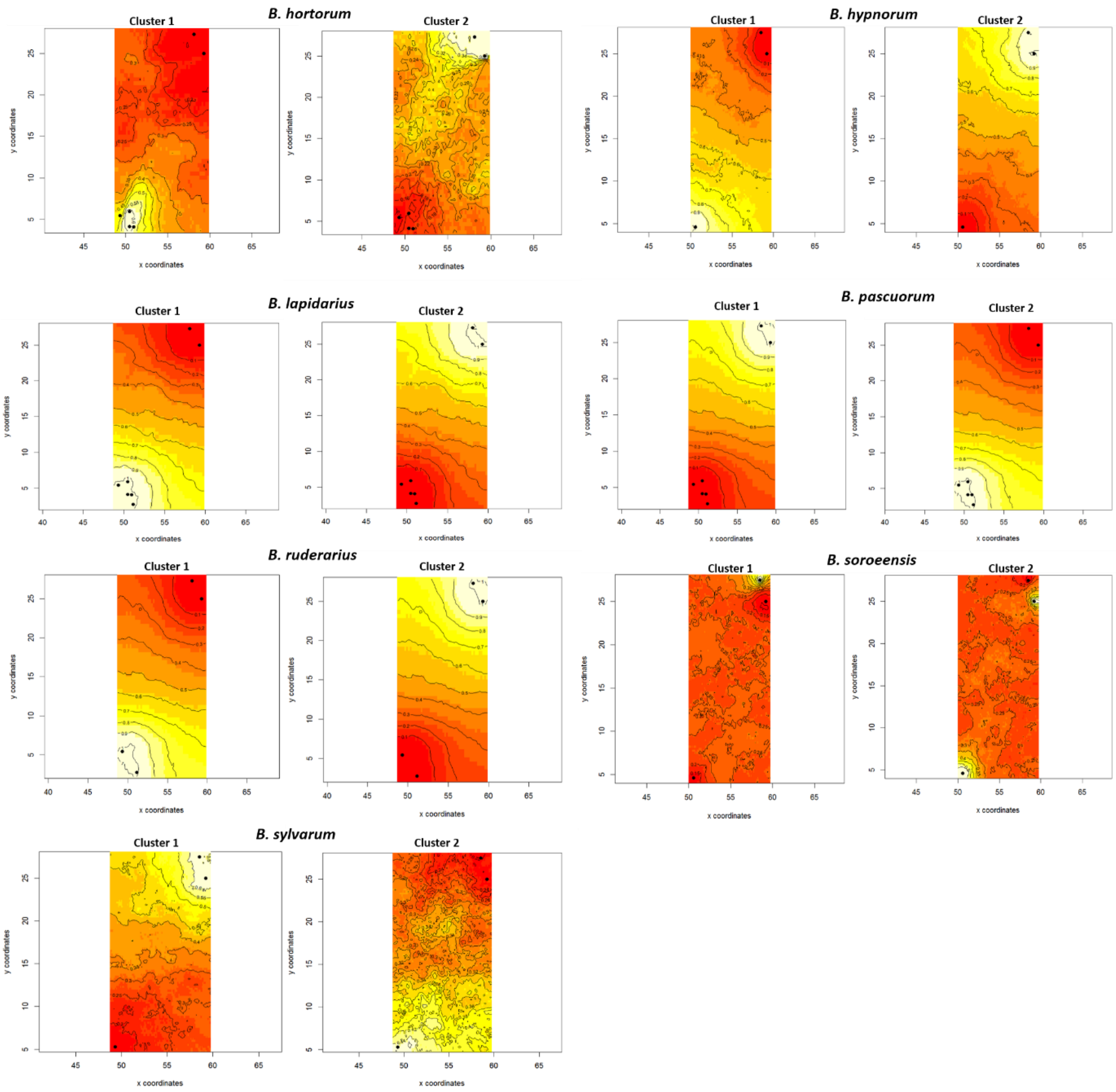

Supplement: Supplementary file 1 — Supplementary datafiles [file 41598_2019_46188_MOESM1_ESM.pdf]
